# Pancreaticoduodenectomy for pancreas carcinoma occurring in the annular pancreas: report of a case

**DOI:** 10.1007/s12328-015-0579-6

**Published:** 2015-07-08

**Authors:** Hiromichi Kawaida, Hiroshi Kono, Mitsuaki Watanabe, Akira Maki, Hidetake Amemiya, Masanori Matsuda, Hideki Fujii, Mitsuharu Fukasawa, Ei Takahashi, Katsuhiro Sano, Tomohiro Inoue

**Affiliations:** First Department of Surgery, Faculty of Medicine, University of Yamanashi, 1110 Shimokato, Chuo, Yamanashi 409-3898 Japan; First Department of Internal Medicine, Faculty of Medicine, University of Yamanashi, Chuo, Japan; Department of Radiology, Faculty of Medicine, University of Yamanashi, Chuo, Japan; Department of Human Pathology, Faculty of Medicine, University of Yamanashi, Chuo, Japan

**Keywords:** Annular pancreas, Pancreas carcinoma, Pancreaticoduodenectomy

## Abstract

The annular pancreas is a rare congenital anomaly in which a ring of the pancreas parenchyma surrounds the second part of the duodenum. Malignant tumors are extremely rare in patients with an annular pancreas. A 64-year-old man presented with appetite loss and vomiting. Abdominal contrast-enhanced computed tomography (CT) indicated pancreas parenchyma surrounding the second part of the duodenum, and a hypovascular area occupying lesion in the annular pancreas. Subtotal stomach-preserving pancreaticoduodenectomy was performed. Histopathology showed pancreatic carcinoma occurring in the complete annular pancreas.

## Introduction

An annular pancreas is a rare congenital anomaly in which the second part of the duodenum is surrounded by a ring of pancreas parenchyma. It was first described by Tiedemann [1] in 1818 and was termed “annular pancreas” by Ecker in 1862 [2]. In adults, annular pancreas has been known to be associated with peptic ulceration, duodenal obstruction, pancreatitis, and obstructive jaundice. The presence of the annular pancreas was reported in three of 20,000 autopsies [3] and three of 24,519 surgical cases [4]. Malignant tumors are extremely rare in patients with an annular pancreas (Table 1). Herein, we report a case of pancreas carcinoma occurring in the annular pancreas.

**Table 1:** Reported cases of an annular pancreas with carcinoma of the pancreas

Case	Author	Age (years)	Gender	Symptoms	Location	Size (cm)	Operation	TNM classification	Outcome (months)	Diagnosis of AP
1	Matsusue [20]	53	F	Abdominal discomfort, spiky fever	Head	3 × 4	TP	IB	15, alive	Laparotomy intraoperation
2	Yasui [20]	54	M	Dark urine, repeated vomiting	Head	2.5 × 3.0	PPPD	IIA	ND	Laparotomy intraoperation
3	Kamisawa [12]	71	F	Epigastralgia	Body	5	Inoperable	IV	4, death	Upper gastrointestinal ERCP
4	Kfir [21]	52	F	Epigastric abdominal pain	Diffuse	ND	TP	IIA	9, death	ERCP
5	Cholet [15]	88	F	Jaundice	Head	4.5 × 2.5	Inoperable	ND	3, death	ERCP, MRCP
6	Present case	64	M	Abdominal pain vomiting	Head (AP)	2.1 × 1.3	SSPPD	IIB	16, alive	CT

*TP* total pancreatectomy, *PPPD* pylorus preserving pancreaticoduodenectomy, *SSPPD* subtotal stomach preserving pancreaticoduodenectomy, *ND* not defined, *ERCP* endoscopic retrograde cholangiopancreatography, *MRCP* Magnetic resonance cholangiopancreatography, *CT* computed tomography

## Clinical summary

A 64-year-old man was admitted to University of Yamanashi Hospital for complaint of abdominal pain and vomiting. On admission, physical examination revealed no anemia and jaundice in the conjunctiva, and no tenderness and no mass in the abdomen. Laboratory findings showed elevated alkaline phosphatases, 898 U/l (100–310), aspartate aminotransferase, 67 U/l (<30 U/l), alanine aminotransferase, 144 U/l (<35U/l) and hemoglobin A1c, 7.7 % (<6.2 %). Furthermore, carbohydrate antigen 19-9 was elevated to 158 U/ml (<37 U/ml); however, carcinoembryonic antigen was within the normal range.

**Fig. 1:**
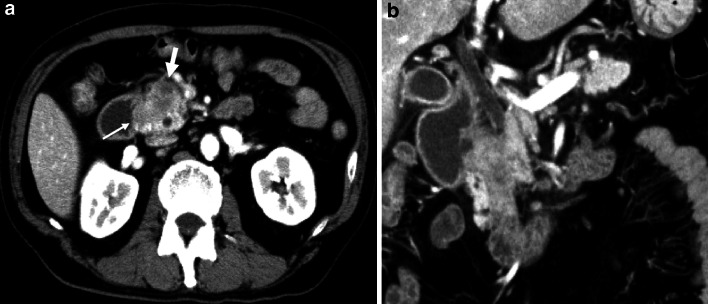
**a** Enhanced CT shows pancreatic parenchyma encircling the descending part of the duodenum (*thin arrow*); and **b** low density lesion is observed in the annular pancreas (*thick arrow*)

**Fig. 2:**
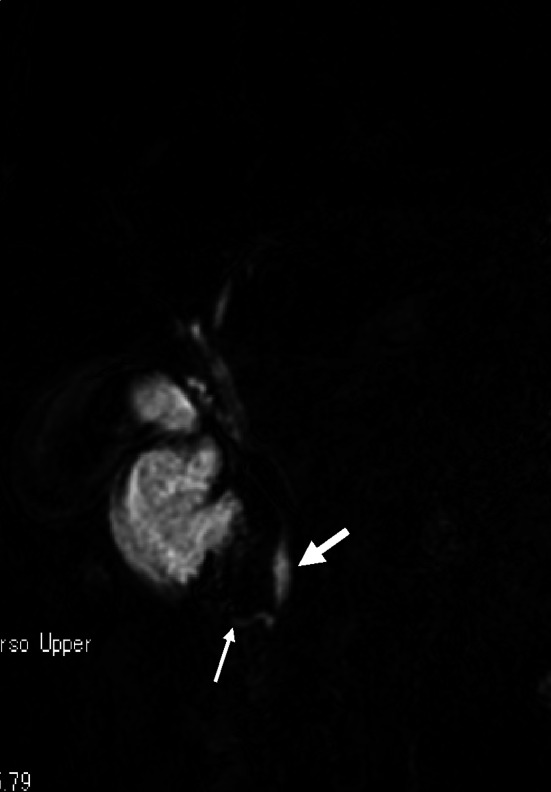
MRCP shows the duct of the annular pancreas (*thin arrow*) connects with the common bile duct (*thick arrow*)

Upper gastrointestinal radiology and esophagogastroduodenoscopy showed stenosis at the descending part of the duodenum. Endoscopic ultrasonography showed a hypoechogenic mass measuring 21 × 13 mm in the pancreas head. In addition, abdominal enhanced-CT revealed the pancreatic parenchyma encircling the descending part of the duodenum, and a hypovascular mass lesion was observed in the annular pancreas (Fig. 1a, b). Magnetic resonance cholangiopancreatography (MRCP) showed the pancreatic duct in the annular pancreas encircling the duodenum connected with the common bile duct (CBD) (Fig. 2). Endoscopic ultrasound-guided fine needle aspiration biopsy (EUS-FNA) was performed and the cytologic findings showed class IV, strongly suspicious of adenocarcinoma.

**Fig. 3:**
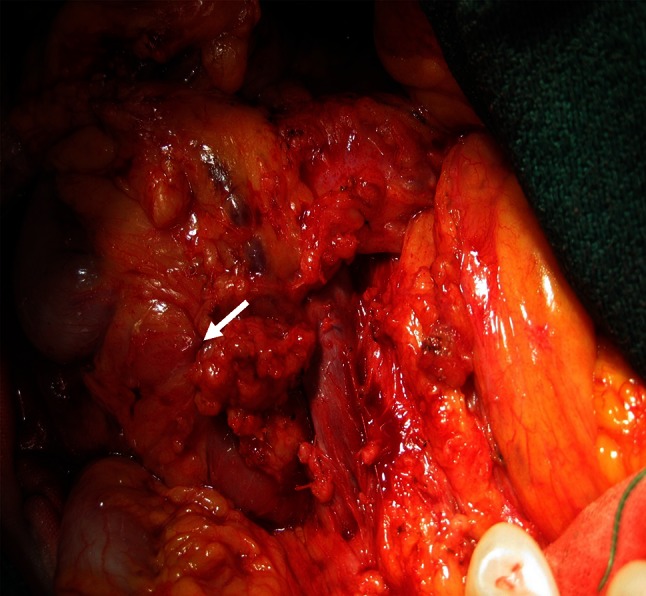
Intraoperative photograph shows the annular pancreas surrounded the descending part of the duodenum (*thick arrow*)

**Fig. 4:**
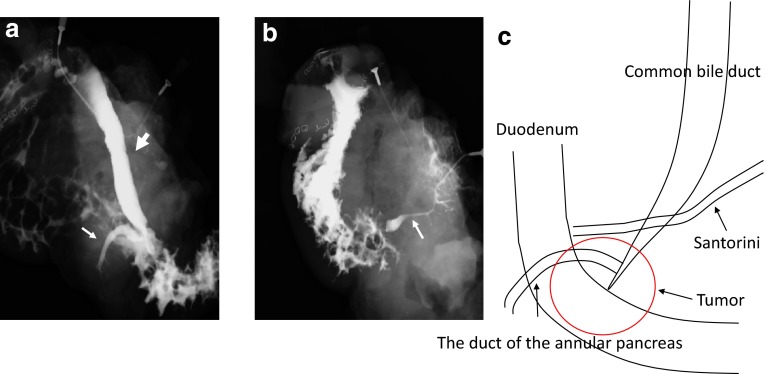
**a** The resected specimen cholangiopancreatography shows the duct of the annular pancreas (*thin arrow*) encircling the duodenum connected with the CBD (*thick arrow*); and **b** could not show the duct of Wirsung (*thin arrow: Santorini*).** c** An illustration

**Fig. 5:**
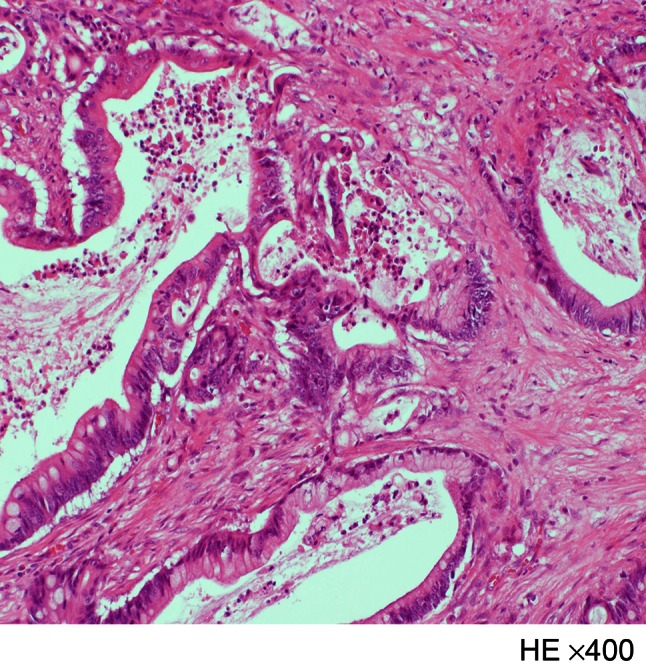
A pathological examination of the resected specimen shows the well-differentiated adenocarcinoma

The patient underwent subtotal stomach preserving pancreaticoduodenectomy with dissection of the regional lymph nodes. At laparotomy, the pancreas parenchymal tissue was surrounding the descending part of the duodenum, and the tumor was located in the annular pancreas (Fig. 3). Neither liver metastasis nor peritoneal dissemination was found. Cholangiopancreatography of the resected specimen showed the pancreatic duct in the annular pancreas, connected to the CBD; it could not show the duct of Wirsung (Fig. 4a, b). A pathological examination showed well-differentiated adenocarcinoma with regional lymph node metastasis (TNM classification: T2N1M0, Stage IIB) (Fig. 5). The postoperative course was uneventful, and the patient was discharged at 26 days after surgery.

## Discussion

The annular pancreas is a rare anatomical anomaly, in which the pancreas parenchyma surrounds the descending part of the duodenum. Most of adult cases become symptomatic at ages between 30 and 50 years [5], and males occupied 65.1 % of total cases [6]. The predominant symptoms of the annular pancreas are epigastric pain, postprandial fullness, vomiting, and weight loss in adults [7, 8]. Furthermore, the main complications of the annular pancreas are peptic ulcers, cholecystolithiasis and pancreatitis [9].

Previously, two hypotheses have been proposed to explain the etiology of the annular pancreas: Lecco’s theory attributes it to adhesion of the right ventral angle to the duodenal wall [10], and Baldwin’s theory considers it to involve persistence of the left ventral bud [11]. Although Lecco’s theory is supported by many reports, not all cases can be explained by only this hypothesis.

The presence of the annular pancreas was reported in three of 20,000 autopsies [3] and three of 24,519 surgical cases [4]. Although the annular pancreas is rare, it has been recently recognized frequently due to progression of diagnostic imaging devices. Among patients undergoing endoscopic retrograde cholangiopancreatography (ERCP), approximately one in 1000 examined cases was found to have the annular pancreas [3, 12]. In these cases, ERCP may not always be successful, due to technical difficulty in some cases, particularly in cases with the duodenal ulcer and/or stenosis of the descending part of the duodenum [13]. In most cases, multi-slice CT shows the circumferential pancreatic parenchyma around the descending part of the duodenum [13, 14]; however, in some cases, this imaging method may lead to misinterpretation as a thickening of the duodenum [15]. With this in mind, magnetic resonance imaging (MRI) and MRCP are useful for understanding anomalies observed in the pancreatic anatomy [13, 15, 16].

The efficacy of endoscopic ultrasonography in the annular pancreas has also been reported, as for less-invasive imaging devices [16, 17]. In addition, EUS-FNA is a highly accurate method for the histological diagnosis of pancreatic carcinoma [18, 19]. In the present case, a definitive diagnosis was possible without operation by EUS-FNA.

Yogi et al. classified six variants of the ductal anatomy in patients with annular pancreas [9]. The most frequent type is type I, in which the main pancreatic duct (MPD) in the annular pancreas opens into the duct of Wirsung. In the second most frequent type, the MPD is encircled the duodenum (type II). The other four types of anomaly are uncommon. The present case is presumed to be type IV, in which the MPD in the annular pancreas opens into the CBD without the duct of Wirsung.

Among the cases reported in the literature, only five have been reported to involve the annular pancreas [12, 15, 20–22]. Among them, three cases were located in the pancreas head, one case in the pancreas body, and one case existed diffusely in the pancreas; however, there were no cases with existing carcinoma in the annular pancreas, except the present case.

In conclusion, to our knowledge, the present report is the first clinical case of pancreatic carcinoma occurring from the annular pancreas. In patients with the annular pancreas, the possibility of coexistent malignancy in the pancreato-biliary system should be considered. EUS-FNA is most likely effective for the definitive diagnosis of pancreatic carcinoma occurring in the annular pancreas. For further elucidation of the annular pancreas, MRCP and the resected specimen cholangiopancreatography would be beneficial.
